# Ectopic taste receptors in animal physiology: evolutionary conservation and functional diversification

**DOI:** 10.3389/fcell.2025.1660529

**Published:** 2025-09-10

**Authors:** Kejin Chen, Xinyu Liang, Hongyu Yi, Guixiang Yu, Qi Wu

**Affiliations:** ^1^ Key Laboratory of Luzhou City for Aging Medicine, Department of Pharmacology, School of Pharmacy, Southwest Medical University, Luzhou, China; ^2^ Central Nervous System Drug Key Laboratory of Sichuan Province, Luzhou, China

**Keywords:** ectopic taste receptor, mammal, teleost, *Drosophila*, nematode

## Abstract

Taste perception is crucial for animals to assess food’s nutritional value while avoiding toxic substances. Recent decades have unveiled the presence of taste receptors beyond the oral cavity, expressed in diverse non-gustatory tissues including gastrointestinal, cardiovascular, reproductive, and neural tissues. These ectopically expressed taste receptors are implicated in a multitude of physiological processes such as the regulation of hormone secretion, nutrient sensing and digestive processes, pathogen defense, and modulation of locomotor abilities. Moreover, these receptors present potential pharmacological targets for therapeutic interventions in diseases related to the respiratory, digestive, and cardiovascular systems. In this review, we summarize the recent advances in understanding the distribution and functions of extraoral taste receptors in mammals, teleosts, insects, and nematodes, emphasizing the commonalities and variations among different species. The emerging paradigm positions taste receptors as polymodal sensors integrating environmental cues with physiological homeostasis beyond their canonical gustatory functions, offering new perspectives on sensory system evolution and organismal adaptation.

## 1 Introduction

Taste perception is an important component of animal adaptation to the environment. Traditionally, it is understood that human can perceive five basic taste modalities: sour, sweet, bitter, salty and umami (Chen et al., 2011; Hoon et al., 1999; Lindemann, 2001). However, certain animals, such as insects, may possess a more diverse array of taste sensations (Chen and Dahanukar, 2020). In mammals, taste is primarily sensed through taste buds confined to the oral cavity (Yarmolinsky, Zuker, and Ryba, 2009), whereas in fruit flies, gustatory organs are more broadly distributed, appearing not only in the mouthparts but also on the legs, wings, and female genitalia (Liman et al., 2014). This broad distribution of taste-related organs is not unique to insects; Similarly, fish have taste organs on their tentacles, fins, and even skin (Ishimaru et al., 2005). While human taste organs are classically considered confined to the oral cavity, the distribution of taste receptors extends into many non-gustatory tissues, contributing to physiological functions beyond taste perception (Behrens and Lang, 2022; Kurumi et al., 2013; Jonas et al., 2019), such as energy homeostasis and immune regulation. These findings have expanded the conceptual framework regarding the roles of taste receptors.

Fruit flies have long served as an important model in taste neurophysiology research, and more recently, studies on piscine taste perception have also emerged as a significant branch of vertebrate taste studies. Despite the extensive distribution of taste organs in these species, the specific roles and distribution of taste receptors in non-gustatory tissues remain inadequately explored. The comparison of ectopically expressed taste receptors’ functions and regulatory mechanisms in non-gustatory tissues across species enables deeper understanding of taste perception’s evolutionary trajectories and provides further insights into the fundamental nature of gustatory mechanisms.

## 2 Taste perception in mammals

**FIGURE 1:**
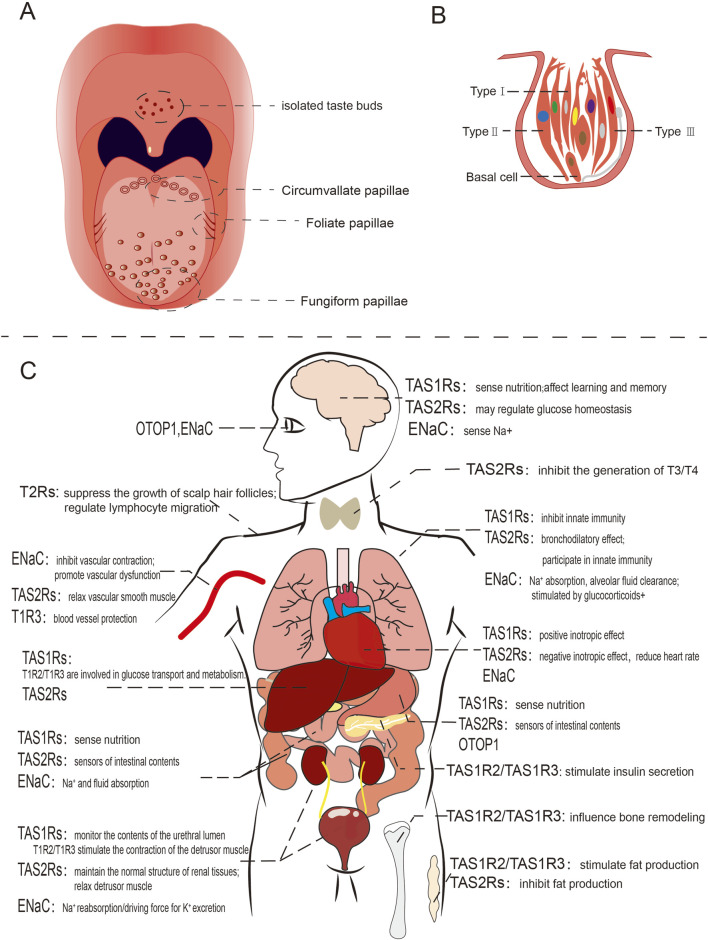
Schematic diagram of human taste organ and ectopically expressed taste receptors. **(A)** The distribution of taste buds on the human tongue; **(B)** Taste bud in humans, direct synaptic connection with afferent nerve fibers showed with gray line; **(C)** Ectopic expression and functions of taste receptors in various human organs.

Human and most other mammals rely on taste buds situated in the oral cavity to discern flavors. These taste buds are embedded within papillae on the tongue’s surface and the palate. They form the basic structure for taste perception. The tongue hosts three types of taste papillae: fungiform, foliate, and circumvallate, while the soft palate contains numerous isolated taste buds (Martin and Martin, 2019; Melis et al., 2020; Doyle et al., 2023; Yarmolinsky et al., 2009) (Figure 1A). It is generally accepted that there are five primary taste modalities:sour, sweet, bitter, salty and umami (Chen et al., 2011; Hoon et al., 1999; Lindemann, 2001; Beauchamp, 2019). However, noncanonical taste qualities such as fat (Brondel et al., 2022; Liu, D, et al., 2018) and alkaline taste have also been identified (Mi et al., 2023). Additionally, other sensory inputs, such as olfactory cues and temperature, can significantly affect taste perception (Talavera et al., 2007; He et al. 2023; Stevenson, 2014). These observations imply the complex combinational logic underlying human taste perception (Fontanini, 2023). In general, various taste stimulations signal the presence of diverse nutrients or toxic substances in the environment, enabling animals to respond. Many foods can trigger multiple taste sensations; for instance, salt can evoke both salty and bitter tastes, depending on its concentration. Moreover, certain bitter foods, like coffee and tea, might ordinarily be expected to elicit aversion, yet they are widely relished (Vecchio et al., 2019; Harwood et al., 2012). This suggests that feeding behavior associated with taste perception, as well as taste perception itself, may be influenced by physiological conditions and even emotional states (Jones et al., 2006; Vincis and Fontanini, 2019).

A human taste bud comprises approximately 50–100 taste receptor cells, which are categorized into four distinct types. Type I cells resembling astrocytes, enclose other cells types, whereas type II and type III cells are elongated and spindle-shaped, featuring taste receptors on their surface. Type III cells have direct synaptic connections with afferent nerve fibers within taste buds. Type IV cells are referred to as basial cells (Doyle et al., 2023) (Figure 1B). The receptors responsible for detecting sweet, umami, and bitter tastes are G-protein-coupled receptors (GPCR), including TAS1R2+TAS1R3 for umami, TAS1R1+TAS1R3 for sweet, and TAS2Rs for bitter (Chen et al., 2011; Ki and Jeong, 2022; Chandrashekar et al., 2000; Grace et al., 2003). In contrast, salty and sour taste are mediated through ion-selective membrane channels (Huang et al., 2006; Chandrashekar et al., 2006; Ishimaru et al., 2006). Each taste is uniquely sensed by its corresponding type of taste receptor cells (Yarmolinsky et al., 2009; Melis et al., 2020; Tomchik et al., 2007).

### 2.1 The taste receptors in mammals

GPCRs (G protein-coupled receptors) are one of the largest families of membrane proteins and are widely involved in various physiological signal transductions. These receptors share a characteristic structure comprising seven transmembrane (TM) helices and interact with heterotrimeric guanine nucleotide-binding proteins (G proteins) (Hu et al., 2017). Human GPCRs can be categorized into several classes: Class A (Rhodopsin) encompasses olfactory receptors, a variety of neurotransmitters receptors, and photoreceptors, comprising approximately 80% of GPCRs. Class B (Secretin) is further subdivided into the secretin family (B1) and the adhesion family (B2) within the GRAFS classification system. Class C (Glutamate) includes metabotropic glutamate (mGlu) receptors, gamma-aminobutyric acid (GABA) receptors, calcium-sensing receptors, and taste receptors. Class F (Frizzled) constitutes another category, while Class T (T2R) is characterized by its low sequence similarity to other classes and complex classification (Herrera et al., 2025; Di Pizio and Niv, 2015). Additionally, there exist other subgroups beyond these primary classes.

**FIGURE 2:**
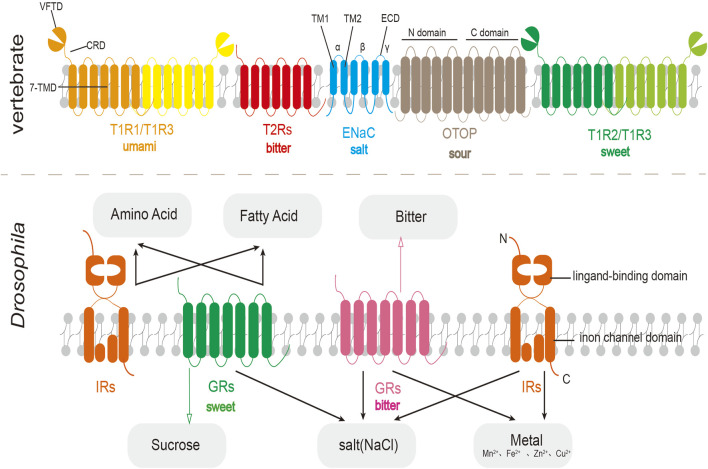
Schematic diagram of the structure of vertebrate taste receptors (top) and fruit fly taste receptors (bottom). In fruit flies, IRs often work together with GRs to perceive taste substances.

The human taste receptor TAS1Rs are members of the Class C GPCRs. TAS1Rs have the typical structural features of this family, consisting of an N-terminal extracellular domain and a seven-transmembrane domain (7-TMD). The N-terminus is further subdivided into a venus flytrap domain (VFTD) and a cysteine-rich domain (CRD). TAS1Rs have a larger bilobed N-terminus and can form a heterodimeric structure (Kim S. K. et al., 2017), for example, the TAS1R2 and TAS1R3 subunits combine to detect sweetness (Charles et al., 2001), whereas the TAS1R1 and TAS1R3 subunits are responsible for umami perception (Grace et al., 2003). Besides, umami taste may also be sensed by the metabotropic glutamate receptor (mGLUR) (Chaudhari et al., 2000; San Gabriel et al., 2005). Bitter taste detection is mediated by the Class T TAS2R family, which similarly contains a seven-transmembrane helix region (7-TMD) (Singh et al., 2011a). Sour taste has been found to be associated with Otopetrin protein (OTOPs) and proteins in the TRP family, specifically PKD2L1/PKD1L3 (Ishimaru et al., 2006). OTOP receptors are homodimers comprising 12 transmembrane helices (TM1–TM12), with TM1–TM6 forming the N-domain and TM7–TM12 forming the C-domain (Saotome et al., 2019). The Epithelial sodium channel (ENaC), responsible for salt taste perception, is a heterotrimeric complex of three distinct subunits: α, β, and γ. Each ENaC subunit consists of two transmembrane domains (TM1 and TM2), with intracellular N- and C-termini and a large extracellular domain (ECD) (Figure 2).

### 2.2 Distribution and function of extraoral taste receptors in mammals

Taste receptors are present in various tissues and organs, including the brain, respiratory tract, lungs, stomach, intestines, pancreas, liver, kidneys, reproductive organs, skin, and muscles, and they are involved in numerous physiological functions (Figure 1C). In this section, we summarize previous research on extraoral taste receptors in mammals. Additionally, we recommend consulting several earlier reviews on this topic for further reading (Behrens and Lang, 2022; Kurumi et al., 2013; Jonas et al., 2019; Li et al., 2024).

#### 2.2.1 Gastrointestinal tract

The anatomical complexity of gastrointestinal tract (GIT) prompts extensive investigation into the differential spatial expression of taste receptors across its segments (Simone et al., 2018; Kok et al., 2018). The distribution and expression levels of bitter taste receptors (TAS2Rs) in the intestine vary among different segments, and different species possess unique expression profiles of these receptors. Studies based on detecting the mRNA expression levels of taste receptors in humans reveals that among the 26 TAS2Rs, TAS2R4 and TAS2R14 are the most ubiquitously expressed subtypes in the GIT (Jalševac et al., 2024). In contrast, certain receptors exhibit strict tissue specificity. TAS2R1, TAS2R42, TAS2R45, TAS2R46, TAS2R50, and TAS2R60 are exclusively detected in the colon, whereas TAS2R7 and TAS2R8 are restricted to the jejunum (Descamps-Solà et al., 2023; Simone et al., 2018).

Rodents (mouse and rat) and human bitter taste receptors do not follow the same expression pattern. Only a few receptors are expressed both in human and rodents’ GIT, including the widely expressed TAS2R4 (homologue of rodent TAS2r118), TAS2R38 (homologue of rodent TAS2r138) expressed in the duodenum, and TAS2R1 specifically expressed in human colon, whereas its rodent ortholog, TAS2r119, is expressed throughout all gastrointestinal tract (GIT) tissues. In addition, two receptors, TAS2R16 and TAS2R41, are not expressed in humans but are expressed in rodents (Descamps-Solà et al., 2023). In mice, sweet taste receptor expression was higher in the ileum than in the jejunum and duodenum (Dyer et al., 2005), whereas umami taste receptors show no significant regional variation in proximal and distal parts of the small intestine (Daly et al., 2013). Despite these findings, the expression landscape of human sweet and umami taste receptors in the GIT remains poorly characterized, highlighting a critical knowledge gap in this field.

**FIGURE 3:**
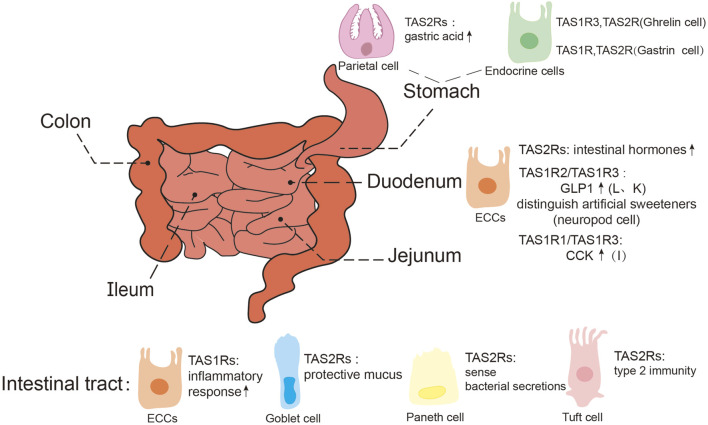
Expression of taste receptors in the gastrointestinal tract. GLP1, glucagon-like peptide 1; CCK, cholecy-stokinin; ECCs, enteroendocrine cells, Including I, K, L cells, etc., are mainly concentrated in the duodenum.

Taste receptors are also expressed in functionally distinct gastrointestinal cells (Figure 3). For example, bitter taste receptors are distributed across various cellular types, including gastric parietal cells, endocrine cells, goblet cells, tuft cells, and paneth cells (Tuzim and Korolczuk, 2021). In gastric parietal cells, TAS2Rs mediate the production of gastric acid (Liszt et al., 2017); within endocrine cells (Kidd et al., 2008; Janssen et al., 2011; Kidd et al., 2009; Kok et al., 2018), these receptors facilitate the release of intestinal hormones such as glucagon-like peptide 1 (GLP1), which play an important role in metabolic regulation (Kok et al., 2018); goblet cells rely on TAS2Rs to promote protective mucus secretion (Prandi et al., 2013), while in paneth cells, these receptors sense bacterial secretions (Simone et al., 2018); and in tuft cells, they are involved in parasite-induced type 2 immunity (Gerbe et al., 2016). In general, TAS2Rs serve as sensors of intestinal contents (Sternini and Rozengurt, 2025). Additionally, in both human and mice gastrointestinal smooth muscle cells, activation of bitter taste receptors also results in inhibition of gastric emptying (Wu et al., 2025; Avau et al., 2015b).

Ectopically expressed sweet taste receptors are primarily located in enteroendocrine cells (Li et al., 2024), particularly L cells and K cells, where the TAS1R2/TAS1R3 receptor complex regulates glucose absorption and GLP-1 secretion. However, it was also reported that the homodimer of TAS1R3 alone is capable of sugar detection (Ohtsu et al., 2014). Studies on mice have revealed that the intestine can distinguish between sugar and artificial sweeteners, with this function predominantly localized to neuropod cells (a type of enteroendocrine cells) in the proximal small intestine rather than the stomach or ileum. Single-cell RT-qPCR analyses have revealed the absence of TAS1R2 expression in neuropod cells, indicating that TAS1R3 is solely responsible for sensing sugars and artificial sweeteners in small intestine (Buchanan et al., 2022). Recent studies have expanded on the role of sweet taste receptors, demonstrating that enteroendocrine cells, through TAS1R3, can detect nutritional factors, triggering inflammatory responses in mice consuming a sugar-rich western diet (Shon et al., 2023). The umami receptors have also been identified in the GIT (Oya et al., 2011; Wang et al., 2011; Daly et al., 2013; Nunez-Salces et al., 2020), contributing to nutritional sensing function (Mace et al., 2009). These findings indicate the multifaceted roles of sweet and umami taste receptors in gastrointestinal physiology.

#### 2.2.2 Cardiovascular system

The expression of bitter taste receptors has been identified in human and mouse cardiomyocytes, as well as in mouse fibroblasts. Activation of TAS2Rs induces a negative inotropic effect (Foster et al., 2014; Foster et al., 2013). Furthermore, these receptors are also present in the sinoatrial (SA) node and left ventricle of rat hearts. Langendorff perfusion of isolated hearts and isolated sinoatrial nodes demonstrated that the activation of bitter taste receptors can reduce heart rate by prolonging ventricular depolarization and attenuating SA node pacing through a phosphodiesterase (PDE)-dependent mechanism (Yuan G, et al., 2020). Notably, each TAS2R encompasses a range of polymorphisms and is highly expressed in cardiac tissue (Foster et al., 2013). Comparative analyses of receptors respond to specific ligands have shown that these polymorphisms can alter receptor signaling. Some result in moderate functional impairment (e.g., TAS2R31 and TAS2R50), while others abolish detectable ligand-dependent signaling (e.g., TAS2R14, TAS2R30, and TAS2R46); conversely, certain variants (e.g., TAS2R10, TAS2R14, and TAS2R31) exhibit slight enhancements in signaling (Bloxham et al., 2024). Receptors with functional alterations are prevalent in the heart and are associated with various physiological and diseases conditions (Foster et al., 2015). These polymorphic variants may influence the feasibility of targeting bitter taste receptors as potential therapeutic interventions for cardiovascular diseases.

TAS1Rs are also expressed in the cardiac tissue, with TAS1R1, TAS1R2, and TAS1R3 localized to the plasma membranes of cardiomyocytes in both mice and humans (Yoder et al., 2025; Papadaki, Osorio- Valencia, and Kirk, 2021). Activation of sweet taste receptors enhances cardiac contractility by exerting positive inotropic effects and accelerating calcium handling (Yoder et al., 2025). Similarly, stimulation of rat ventricular myocytes with umami agonists has been shown to induce comparable inotropic responses (Papadaki et al., 2021). These findings suggest that sweet and umami taste receptors function as nutrient sensors, modulating cardiac contractility in response to metabolic cues. Given their regulatory role, TAS1Rs may hold therapeutic potential for conditions such as heart failure, warranting further investigation into their clinical applications.

There are also taste receptors on blood vessels. Activation of bitter taste receptors induces relaxation of vascular smooth muscle cells (Manson et al., 2014). These receptors have been identified in various vascular beds, including the aorta of mice (Manson et al., 2014), pulmonary arteries (Manson et al., 2014) and omentum arteries of humans, as well as the mesenteric and cerebral arteries of rats (Chen et al., 2017). The sweet receptor TAS1R3 is expressed in the endothelial cells of human primary glomerular microvessels (Enuwosa et al., 2021), human retinal microvessels (Lizunkova et al., 2019), and rat pulmonary microvessels, where it exerts a protective effect on blood vessels. In contrast, The ENaC is expressed in endothelial cells, where exposure to high salt concentrations promotes vascular dysfunction, characterized by reduced nitric oxide production, increased cell stiffness, and pressure-independent vascular remodeling (Mutchler et al., 2021; Zheng et al., 2016). ENaC is also found in smooth muscle cells, inhibition of this channel effectively nullifies pressure-induced vascular contraction in the renal arteries of mice (Jernigan and Drummond, 2005) and cerebral vessels of rats (Kim et al., 2012). It is plausible that taste receptors are present in additional regions of the cardiovascular system, such as the coronary arteries, which supply blood to the heart and whose dysfunction may result in angina pectoris (Savulescu-Fiedler et al., 2025). Investigating the roles and expression patterns of taste receptors in these arteries could provide valuable insights into developing novel therapeutic strategies for angina pectoris.

#### 2.2.3 Reproductive system

**FIGURE 4:**
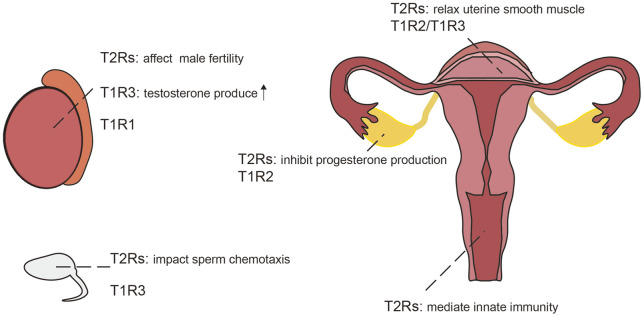
Expression of taste receptors in the reproductive system,the testicles and sperm of the male (left); the reproductive system of the female (right).

Taste receptors are also present in the reproductive system and play critical roles in maintaining male and female fertility as well as regulating immune function within this system (Lu et al., 2024) (Figure 4).

##### 2.2.3.1 Testis and sperm

TAS2r105 is expressed in early to mid-stage round spermatids in mice, but not present in spermatocytes or spermatogonia (Li and Zhou, 2012). In contrast, TAS2r131 is distributed throughout the lumen of seminiferous tubules of adult testes and small tubules in the epididymis in 3-day-old mice (Voigt et al., 2015; Voigt et al., 2012). Recent studies have further corroborated the presence of TAS2Rs subtypes and their downstream signaling components in human testes and sperm, suggesting a conserved role across species (Governini et al., 2020). Bitter taste receptors are increasingly recognized as significant contributors to physiological processes such as sperm chemotaxis and male fertility (Lu et al., 2024), similarly, some olfactory receptors also involved in the sperm chemotaxis process (Spehr et al., 2003). Besides bitter taste receptors, sweet and umami taste receptor subunits TAS1R1 and TAS1R3 have also been identified in both human and murine testes (Frolikova et al., 2020; Meyer et al., 2012), with TAS1R3 specifically located on the head of sperm in mice (Frolikova et al., 2020). Stimulation of TAS1R3 enhances testosterone production in Leydig cells via the cAMP-PKA-SP1 pathway (Liu et al., 2025; Shon et al., 2024; Liu et al., 2022), where TAS1R3 knockout results in reduced male fertility (Seong et al., 2024; Shon et al., 2024). In addition, abnormal spermatogenesis was found in mice deficient in the umami receptor subunit TAS1R1 (Meyer et al., 2012).

##### 2.2.3.2 Ovaries, uterus, placenta and vagina

Bitter taste receptors are also present in the female reproductive system. Specifically, TAS2Rs and their downstream signaling components been identified in granulosa cells and cumulus cells within the human ovary (Semplici et al., 2021). Experiments using high concentrations of saccharin to activate TAS2Rs in the corpus luteum of rats have demonstrated an inhibition of progesterone production in pseudopregnant rats, mediated through NO/cGMP signaling and apoptosis pathways (Jiang et al., 2021). The sweet taste receptor TAS1R2 is similarly expressed in the corpus luteum of the rat ovary (Jiang et al., 2021). Administration of sweet taste agonists has been shown to modulate ovarian function, with saccharin increasing and stevia decreasing progesterone levels in young rats (Jiang et al., 2018); However, dietary effects cannot be entirely ruled out as contributing factors. Similar to saccharin, certain steviosides can also activate bitter taste receptors in gustatory organs (Tian et al., 2022); but whether ectopically expressed bitter taste receptors in the reproductive system can likewise be activated by steviosides remains to be further investigated.

Transcripts of bitter taste receptors and their associated signal transduction components have been detected in the myometrium of both human and mice. Activation of bitter taste receptors with chloroquine induces relaxation of precontracted uterine smooth muscle strips, outperforming currently used tocolytics (Zheng et al., 2017). Moreover, activation of TAS2R5 with phenanthroline leads to extensive uterine relaxation (Delpapa et al., 2023), suggesting potential as novel uterine relaxants. Sweet taste receptor, TAS1R2/TAS1R3, have also been identified in the uterus of guinea pigs (Li et al., 2020), although their precise role within the uterine context warrants further investigation.

The TAS2R14 gene has been detected in human and mouse placentas, as well as in human trophoblast cell lines (Taher et al., 2019). TAS2R38 is expressed in human placental tissue and JEG-3 cells, where it demonstrates functionality by facilitating calcium influx and potentially mediating innate immune response to placental infection (Wölfle et al., 2016). Furthermore, TAS2r143 was found in epithelial cells of the vagina and cervix in mice and may also be involved in innate immunity (Liu et al., 2017).

#### 2.2.4 Respiratory system

The expression of TAS2R38, TAS2R4 and TAS2R16 proteins has been identified in ciliated cells of human sinus epithelium. Notably, TAS2R4 and TAS2R16 mediate NO-dependent immune defenses, leading to an increase in ciliary beat frequency, which accelerates the clearance of nasal mucosa cilia (Yan et al., 2017).

Tuft cells, also known as solitary chemosensory cells (SCC), are found in epithelial tissues at various sites, including the nasal mucosa of the upper airway and lower airway (Ye and Bankova, 2022). In rat and mouse sinonasal SCC, both mRNA and protein levels of TAS2Rs and TAS1R3 expressions can be detected (Tizzano et al., 2011). Another study using immunofluorescence techniques has demonstrated the presence of TAS2Rs and TAS1R2/TAS1R3 in human sinonasal SCCs, indicating that TAS2Rs are widely distributed throughout the respiratory epithelium and may play a role in innate immunity (Lee R. J. et al., 2014). In contrast, sweet and umami taste receptors within the respiratory epithelium have been observed to inhibit the innate immunity mediated by bitter taste receptors under both healthy and inflammatory conditions (Lee R. J. et al., 2014; McMahon et al., 2023a, 2023b).

In both mice and humans, the activation of TAS2R receptors induces relaxation of bronchial smooth muscle (Deshpande et al., 2010; Grassin-Delyle et al., 2013). Importantly, the relaxation effect induced by bitter taste receptors is similar to that of the β2 agonist isoproterenol (Deshpande et al., 2011), without functional downregulation during airway inflammation (Robinett et al., 2014), and maintaining full efficacy during β2 agonist tolerance (An et al., 2012). These findings suggest that TAS2R receptor agonists could serve as potent bronchodilators.

#### 2.2.5 Brain

Taste receptors are widely distributed in the mammalian brain. Transcripts and proteins of TAS2Rs have been detected in the brainstem, cerebellum, cerebral cortex and nucleus accumbens of rats (Singh et al., 2011b), as well as in the epithelial cells of the human choroid plexus (CP), with *in vitro* studies demonstrating responsiveness of human CP epithelial cells to bitter tastants (Duarte et al., 2020). While numerous exogenous bitter compounds are blocked by the blood-brain barrier (BBB), certain food-derived dipeptides and tripeptides have been shown to cross the BBB, though their physiological effects remain unclear (Upadhyaya et al., 2010). As an active interface between blood and cerebrospinal fluid (CSF), the choroid plexus may utilize taste receptors to mediate the sensing of blood-borne substances. Notably, TAS2R14 has been implicated in modulating the activity of ABC efflux transporters in the CP, thereby regulating the transcellular transport of resveratrol across the human blood-CSF barrier (Duarte et al., 2020). Recent studies have identified a subset of functional tanycytes (neuroglia-like cells) in the median process at the base of the brain, which do not express sweet taste receptors and whose ablation leads to impaired glucose tolerance, suggesting that bitter taste receptors may be involved in the regulation of glucose homeostasis (Yu et al., 2023). In addition, psychotropic drugs exposure alters cerebral bitter taste receptors expression, indicating these receptors as potential pharmacological targets (Ansoleaga et al., 2015).

The sweet taste receptor TAS1R2 is ubiquitously present across various neuronal populations in mice, with localization in glial cells of periventricular organs and vascular structures within the cortex, thalamus, and striatum (Jang et al., 2021). Intriguingly, while a subset of tanycytes express bitter taste receptors, the majority exhibit TAS1R2/TAS1R3-mediated glucose sensing (Benford et al., 2017; Kohno et al., 2016). Genetic ablation of TAS1R3 in mice leads to changes in learning and memory functions and deficits in social ability, underscoring its critical role in central nervous system neurotrophic functions (Martin et al., 2017).

#### 2.2.6 Other organs and systems

In the immune system, bitter taste receptors are expressed in various human immune cells, including macrophages, lymphocytes, monocytes and neutrophils (Gopallawa et al., 2021; Tran et al., 2018; Maurer et al., 2015; Malki et al., 2015),regulating innate and adaptive immunity and suppressing the production of inflammatory factors. Umami taste receptors (TAS1R1/TAS1R3) in mouse neutrophils mediate chemotaxis and attenuate lipopolysaccharide-induced inflammatory responses upon activation (Lee N et al., 2014).

The urinary system demonstrates taste receptor-specific modulation. TAS2Rs in murine kidneys (Rajkumar et al., 2014; Liu et al., 2015) and human bladders (Zhai et al., 2016) maintain glomerular structure and promote detrusor relaxation, whereas sweet taste receptors (TAS1R2/TAS1R3) induce detrusor contraction in rat bladders (Elliott et al., 2011). Adipose tissue expresses both receptor families across species (Cancello et al., 2020; Avau et al., 2015a; Simon et al., 2013), and exhibiting significant roles in adipogenesis regulation (Avau et al., 2015a; Ning et al., 2016), but the underlying mechanisms are controversial (Ki and Jeong, 2022; Tuzim and Korolczuk, 2021). Expression of TAS1Rs and TAS2Rs has also been found in mouse liver (Kurtz et al., 2020; Liu S, et al., 2018), where TAS1R2/TAS1R3 are involved in glucose transport and metabolism (Luo et al., 2022). Moreover, pancreatic β-cell TAS1R2/TAS1R3 mediate sweetener-induced insulin secretion (Nakagawa et al., 2009), while influencing bone remodeling processes (Simon et al., 2014). Notably, TAS2Rs exhibit pleiotropic effects, modulating inflammation in mouse gingival SCC(Zheng et al., 2019), inhibiting thyroid hormone (T3/T4) secretion in thyrocytes (Clark et al., 2015), and suppressing hair follicle growth in human scalp tissue (Sakakibara et al., 2022; Gherardini et al., 2025).

Beyond classical taste receptors, ENaC regulate fluid homeostasis in pulmonary, colonic, renal, and ocular tissues (Rotin and Staub, 2021). Free fatty acids receptors (FFARs) demonstrate broad tissue distribution including CNS and pancreatic expression (Roy et al., 2022; Kimura et al., 2019), while OTOP1 localizes to brown adipose tissue and reproductive organs (Tu et al., 2018), though their precise physiological mechanisms remain incompletely characterized.

Collectively, ectopically expressed taste receptors across various tissues and organs have been implicated in numerous non-gustatory functions. Unlike canonical taste perception, these functions mediated by ectopic taste receptors, such as the chemotaxis of immune cells and sperm, can operate independently of complex neural circuitry. Consequently, the conventional boundaries demarcating the operational paradigms of taste receptors from other chemosensory receptors, as well as their respective physiological roles, have become increasingly blurred. This phenomenon finds parallel observations in studies of teleosts and invertebrates (see subsequent sections).

## 3 Taste perception in teleosts

**FIGURE 5:**
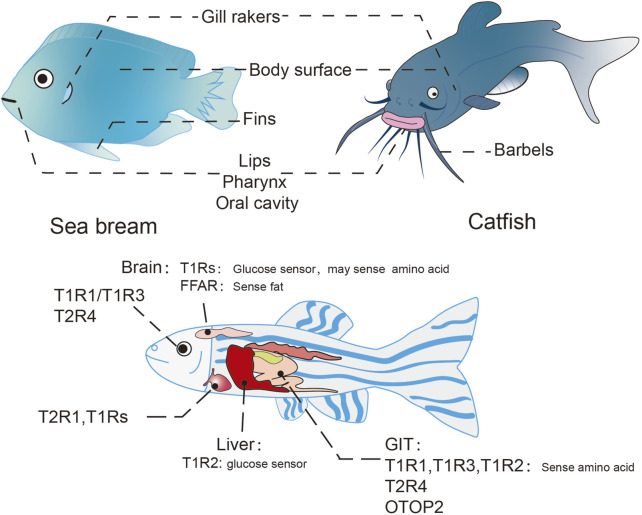
Taste organ and ectopically expressed taste receptors in teleosts. Distribution of taste organs in teleost (above), taking the sea bream and catfish for example; Ectopic taste receptors in teleost (below), taking the zebrafish for example.

Teleost fishes represent a diverse group of vertebrate species, with zebrafish (*Danio rerio*), medaka (*Oryzias latipes*), and pufferfish (*Tetraodon nigroviridis*) serving as predominant model organisms in scientific research (Yasuoka and Abe, 2009). The gustatory system in these species exhibits a broad anatomical distribution, with taste buds localized in the lips, gill rakers, pharynx, oral cavity, and also on integumentary surface, while some species additionally possess these sensory structures on barbels and fins (Ishimaru et al., 2005; Hardy and Hale, 2022; Ovalle and Shinn, 1977; Yacoob et al., 2001) (Figure 5). Morphologically analogous to mammals, piscine taste buds display elongated, onion-shaped or pear-shaped configurations, embedded within sensory epithelium richly innervated by afferent nerve fibers. These chemosensory organs comprise three distinct cell populations: taste receptor cells (TRCs), supporting cells, and basal cells (Morais, 2017; Giaquinto et al., 2024). Despite the extensive utilization of teleost models in biological research, investigations of gustatory mechanisms remain relatively underdeveloped (Okada, 2015). Systematic studies of taste perception pathways in fish have only emerged in recent years. Comparative analyses across multiple species reveal subtle interspecific variations in both the spatial distribution of taste buds and the expression patterns of taste receptors (Yuan X. C. et al., 2020; Hardy and Hale, 2022; Bhatia et al., 2022; John et al., 1993; Maik, Tatjana, and Sigrun, 2023).

Marine animals and terrestrial animals differ in the mechanism of the “tasting” behavior. For terrestrial animals, tasting typically involves the direct contact with substances like sugar or amino acids which are non-volatile and water-soluble. In contrast, marine animals, immersed in an aqueous environment, can perceive those substances from a distance, in a manner functionally analogous to olfaction in terrestrial animals. Conversely, certain volatile, water-insoluble substances that are perceived through smell on land can be perceived by marine animals through direct contact, similar to the “taste” mechanism in terrestrials (Mollo et al., 2014; Giordano et al., 2017; Mollo et al., 2022). Therefore, when studying the “taste” receptors of animals (especially marine animals), one should not be confined to the traditional binary classification framework of “smell-taste”. An open research perspective should be maintained to avoid ignoring potential ligand substances.

### 3.1 The taste receptors in teleosts

Comparative genomic analyses reveal that piscine T1R and T2R taste receptors exhibit sequence homology with their mammalian orthologs. T1R1 and T1R3 demonstrate high evolutionary conservation across fishes and mammals, whereas fish T1R2 shows only weak homology to the mammalian T1R2 genes (Okada, 2015; Ishimaru et al., 2005). While vertebrates T1R1/T1R3 heterodimer typically function as umami receptors and T1R2/T1R3 as sweet receptors (Yarmolinsky et al., 2009; Yuan X. C. et al., 2020), teleost fishes exhibit a distinctive adaptation: both T1R1/T1R3 and multiple T1R2/T1R3 variants serve as amino acid chemoreceptors. The evolutionary advantage of maintaining multiple amino acid taste receptors remains unclear, though it may reflect an adaptive strategy to diverse dietary niches, given the critical role of amino acids in energy metabolism for both carnivorous and herbivorous species (Calo et al., 2021).

The number of T2R genes among vertebrates exhibits significant variability; for instance, most fish possess merely 2 ∼ 5 T2Rs, whereas mammals typically have approximately 20 ∼ 50 T2R genes (Dong et al., 2009). This diversity arises through dynamic birth-and-death evolutionary processes that generate both lineage-specific functional subtypes and nonfunctional pseudogenes (Nei and Rooney, 2005). The bitter taste system is widely hypothesized to have evolved as a chemosensory defense mechanism, with T2R gene family expansion patterns showing strong correlation with ecological niche specialization and dietary adaptation. The remarkable expansion observed in certain lineages likely reflects the selective pressures imposed by complex phytochemical environments (Dong et al., 2009; Shi and Zhang, 2006). The substantial interspecies variation in the repertoire and diversity of T2R receptors results in marked differences in both the recognition spectrum and sensitivity to bitter compounds among species. A substance perceived as bitter by humans does not necessarily elicit similar responses in other species, as this depends on the presence of specific T2R receptors capable of detecting the compound. For instance, certain substances classified as ‘extremely bitter’ by human standards fail to evoke significant avoidance behaviors in fish, indicating that bitter substances have specificity for different species (Shi and Zhang, 2006; Oike et al., 2007; Meyerhof et al., 2010; Kasumyan and Døving, 2003).

Fish also possess specialized sensory mechanisms including acid-sensing ion channels (ASICs) and transient receptor potential (TRP) channels (Montalbano et al., 2021; Levanti et al., 2016). As aquatic organisms, osmoregulation presents unique challenges, particularly for freshwater species. Intriguingly, while mammals utilize ENaC for salt detection, zebrafish and other teleosts appear to have evolved alternative salt-sensing pathways, potentially involving olfactory integration, given their apparent lack of ENaC-mediated salt taste transduction (Herrera et al., 2021).

Phylogenetic comparison of taste receptor evolution provides valuable insights into the adaptive logic of sensory system diversification. However, significant gaps remain in our understanding of ectopic taste receptor evolution, particularly regarding the conservation of non-gustatory functions across species. Further investigation of these receptors may uncover fundamental principles governing the functional diversification of chemosensory systems during vertebrate evolution.

### 3.2 Distribution and function of ectopic taste receptors in teleosts

Emerging evidence demonstrates widespread expression of taste receptors in non-gustatory tissues of teleost fishes, including the gastrointestinal tract, visual system, cardiovascular organs, and hepatic tissues (Yuan X. C. et al., 2020; Xia et al., 2020; Yanan et al., 2017) (Figure 5). However, these ectopic chemosensory receptors remain poorly characterized across the phylogenetic diversity of fish species, warranting systematic investigation of their spatiotemporal expression patterns and functional divergence.

#### 3.2.1 Gastrointestinal tract

Comparative analyses reveal distinct organizational principles of taste receptor distribution in the teleost gastrointestinal tract compared to mammalian systems. In rainbow trout, T1R1 exhibits pan-enteric expression, while two T1R2 paralogs show segment-specific enrichment, demonstrating elevated expression in gastric mucosa and distal intestinal regions. Conversely, T1R3 maintains constitutive but low-level expression throughout the gastrointestinal epithelium (Calo et al., 2021). Another study in snapper reflected a significantly higher distribution of T1R3 in the midgut and hindgut segments (Angotzi et al., 2022), suggesting conserved yet species-specific patterns of amino acid chemoreception along the enterohepatic axis. These observations support a model of compartmentalized nutrient sensing, where persistent luminal amino acid detection in distal segments may compensate for incomplete digestive processes. *In situ* hybridization analyses reveal both autonomous and enteroendocrine cell (EEC)-associated localization of T1Rs in snapper, suggesting that T1Rs-mediated chemosensing may occur in these tissues through an endocrine dependent and independent mechanism (Angotzi et al., 2022).

Other receptors, such as the acid-sensing receptor OTOP2 also express in myenteric neuronal subsets of the enteric nervous system as well as in EECs (Levanti et al., 2011), suggesting integrated chemosensory-neuroendocrine circuits in teleost gut physiology.

#### 3.2.2 Brain

Multiple T1R genes have been identified as being expressed in the brain of the snapper, with T1R2d showing significantly high levels of expression in both the forebrain and hindbrain compared to other T1R genes, and T1R2b being markedly elevated in the midbrain (Angotzi et al., 2022). T1Rs have been found to be involved in nutrient perception in the brain, in which the hypothalamus plays an important role (Soengas et al., 2024). In rainbow trout (Otero-Rodiño et al., 2015) and mammals (Ren et al., 2009), the exposure of the hypothalamus to glucose resulted in decreased expression of T1R2/T1R3, suggesting a potential role in glucose sensing. Contrastingly, another study revealed that glucose stimulation in the telencephalon, a part of the forebrain in snapper, resulted in an upregulation of T1R2/T1R3 expression. This discrepancy implies that taste receptors in the telencephalon could be engaged in glucose-dependent processes that function as a reward mechanism (Cristina et al., 2018). Studies into amino acid sensing pathways in rainbow trout (Comesaña et al., 2017; Comesaña et al. 2020; Comesaña et al. 2024) indicates an active amino acid sensing mechanism within the hypothalamus, potentially involving umami receptors, and demonstrates that amino acid stimulation in the ventricle can affect both central and peripheral metabolic processes. Furthermore, adipose receptors FFA1 and FFA4, also expressed in the hypothalamus, are implicated in fat sensing and participating in the regulation of food intake (Velasco et al., 2020). Additional chemosensory components, including OTOP channels, are broadly expressed throughout the central nervous system, though their precise functional contributions remain to be elucidated (Paukert et al., 2004).

#### 3.2.3 Other organs and systems

In rainbow trout, T1Rs and T2R4 have been identified in the mucosal tissues of various regions, including the eye mucosa; however, the function of taste receptors in ocular tissue remains undetermined (Xia et al., 2020). T1Rs have also been detected in the liver of grass carp (Yuan X. C. et al., 2020), with *in vitro* studies on rainbow trout liver suggesting that the sweet taste receptor may function as glucose sensor (Otero-Rodiño et al., 2016). Cardiac tissues likewise exhibit T1R expression, indicating possible chemosensory functions in cardiovascular regulation that await mechanistic characterization (Roy et al., 2022).

Research on the common carp (Toshiki et al., 2021) identified three non-gustatory T2R isoforms, ccT2R200-2, ccT2R201, and ccT2R203-1, that respond to bitter compounds, implying novel chemosensory roles in systemic physiology. Nonetheless, the exact *in vivo* distribution and functions of these genes require further investigation. In zebrafish, RL-TGR, an aversive taste co-receptor, has been detected in the mechanosensory cells of neuromasts within the lateral line. Disruption of RL-TGR activity adversely affects normal lateral line development (Mojib et al., 2017). Previously, it has been shown that the chemokine receptor Cxcr4, which is mediated by GPCR signaling, plays a crucial role in the primordial cell migration in the lateral line (Yu et al., 2012; Dambly-Chaudière et al., 2007). Therefore, RL-TGR, alongside unidentified GPCRs, may significantly impacts the development of the lateral line system.

## 4 Taste perception in *Drosophila*

*Drosophila melanogaster* exhibits a taste perception spectrum comparable to that of mammals, capable of detecting the five canonical taste modalities (Yarmolinsky et al., 2009; Liman et al., 2014) alongside alkaline (Mi et al., 2023), lipid (Tauber et al., 2017), and heavy metal taste sensations (Li X, et al., 2023). Notably, *Drosophila* demonstrates exceptional gustatory sensitivity, with behavior studies revealing sucrose detection thresholds as low as 0.5 mM - an order of magnitude more sensitive than human perception (5 mM threshold). Furthermore, flies can detect B vitamins at nanomolar concentrations (10 nM) (Wu et al., 2021; Weiffenbach et al., 1982). Despite the similarities in detectable taste types between flies and mammals, their peripheral neural architectures differ fundamentally: mammalian taste buds contain gustatory receptor neurons (GRNs) capable of responding to all taste categories (Liman et al., 2014), whereas *Drosophila* employs distributed sensory processing through distinct GRN combinations across spatially segregated taste bristles (Makoto et al., 2002).

### 4.1 An overview of taste organs in *Drosophila*

**FIGURE 6:**
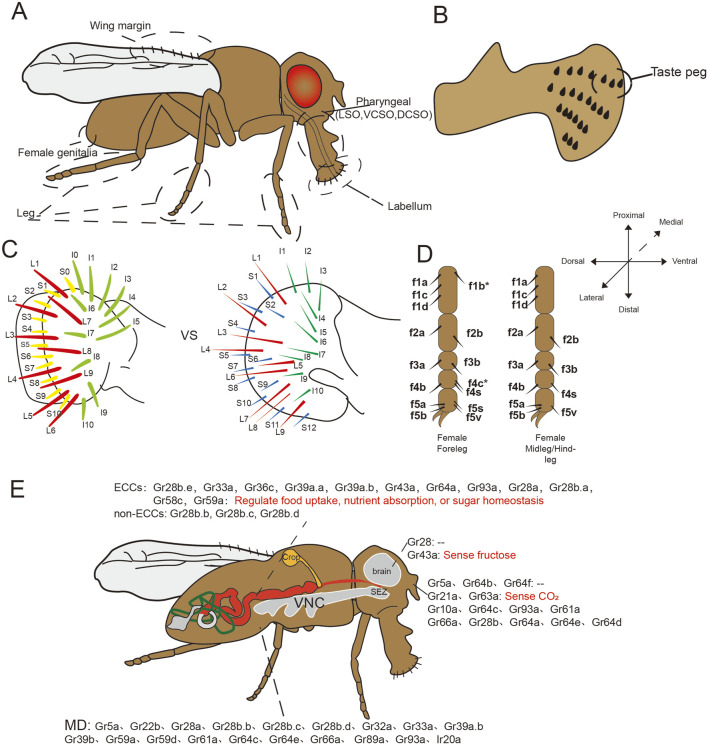
Schematic diagram of taste organs and ectopic expression of taste receptors in *Drosophila*. **(A–D)** The taste organs of *Drosophila* include the labellum, legs, wing margins, and even the female genitalia as external taste organs, the pharynx as an internal taste organ; (C) Comparison of the two distribution pattern of *Drosophila* labellar bristles in *Canton S* strain, the left side (Linnea et al., 2011) has one less S-type bristle and one more L-type bristle compared with the right side (Makoto et al., 2002); **(E)** Ectopic expression and functions of taste receptors in fruit flies. SEZ, subesophageal zone; VNC, ventral nerve cord; MD, multidendritic neurons.

The taste organs of *Drosophila* are distributed across multiple body parts, with external organs located on the proboscis, the anterior margins of the wings, the distal five segments of the legs, and the female ovipositor (Figure 6A). Complementing these external structures, three internal pharyngeal taste organs are present: the labral sense organ (LSO), the ventral cibarial sense organ (VCSO), and the dorsal cibarial sense organ (DCSO) (Chen and Dahanukar, 2020; Liman et al., 2014; Stocker, 1994; Chen and Dahanukar, 2017). In adult *Drosophila*, the proboscis, analogous to the mammalian tongue, terminates in two fused labella serving as an external tasting apparatus. Each labellum hosts 31 taste bristles, categorized by morphology into S-type (short), I-type (intermediate), and L-type (long). L-type bristles, distinguished by their length, predominantly sense attractive flavors, lacking neurons responsive to bitter taste. In contrast, I-type bristles comprise neurons for sweet and bitter tastes, while S-type encompass four neuronal types: A: responsive to sugar and low sodium concentrations; B, reacting to aversive chemicals such as bitter compounds; C, detecting water; and D, responding to calcium and elevated cation concentrations. Approximately 30 taste pegs are situated on the inner surfaces of each labial palp (Figure 6B), with about six dedicated to sugar detection and the remainder responsive to CO_2_(Jaeger et al., 2018; Singh, 1997; Meunier et al., 2003; Yarmolinsky et al., 2009).

Notably, interstrain variation exists in labellar bristle number, with the *Canton S* strain consistently exhibiting 31 bristles per labellum. However, two distinct bristle distribution patterns have been documented even within this strain, one pattern displays an additional S-type bristle and one less I-type bristle compared to the other (Figure 6C) (Isono and Kikuchi, 1974; Raphael et al., 1976; Krishanu et al., 1993; Shubha et al., 2001; Makoto et al., 2002; Linnea et al., 2011).

In neurophysiological analyses of *Drosophila* leg taste bristles, the female foreleg is typically used as a representative model due to its heightened density of sensilla, including unique f5s sensilla absent from other legs (Shubha et al., 1983; Ling et al., 2014; Kopp and Barmina, 2022) (Figure 6D). These sensilla facilitate gustatory engagement as fruit flies often utilize their forelegs for tasting. Relative to other legs, more foreleg sensilla respond to sugars, and these sensilla exhibit heightened sensitivity to bitter compounds (Ling et al., 2014). Despite these findings, comparative analyses of receptor expression across different legs remain limited, with available data primarily derived from taxa such as nymphalid butterflies and the blowfly *Phormia regina* (Ômura et al., 2011; Dethier and Vincent, 1976). The anatomical positioning of mid and hindlegs presents challenges to electrophysiological analysis. This knowledge gap presents intriguing questions regarding potential leg-specific taste processing, including differential sensitivity to identical stimuli across appendages or integration of conflicting foreleg/midleg inputs. Furthermore, the functional characterization of other gustatory structures including wing, genital, and pharyngeal organs remains incomplete, representing promising avenues for future research (Chen and Dahanukar, 2020).

### 4.2 The taste receptor in *Drosophila*

The taste receptors of *Drosophila* primarily consist of the gustatory receptor (GR) family, which comprises 68 known seven-transmembrane proteins (Arntsen et al., 2024). Initially believed to be exclusive to invertebrates, recent evidence suggests that these receptors originated from a common eukaryotic ancestor. Moreover, two additional lineages have been identified, demonstrating that its superfamily has not been completely lost in chordates (Benton and Himmel, 2023). Sweet taste receptors, such as Gr64f, are primarily responsible for detecting attractive substances like sucrose, while bitter taste receptors, exemplified by Gr66a, perceive aversive substances including caffeine. Other key taste receptor families include the Ionotropic receptor (*Irs*), which detect Na^+^, fatty acid, amino acid, and heavy metal ions; the *pickpocket* (*ppk*) receptors, which respond to water (*ppk28*) and pheromone (*ppk23*); and *Transient receptor potential* (*Trp*) receptors, implicated in sensing noxious or pungent compounds (Chen and Dahanukar, 2020; Shrestha and Lee, 2023; Freeman and Dahanukar, 2015). *Irs*, which are critical and ubiquitously expressed in fruit flies, share structural characteristics with synaptic, glutamate-gated ion channels, featuring three transmembrane domains and a bilobed extracellular binding domain (Arntsen et al., 2024) (Figure 2).

In recent years, investigations into the neurophysiological mechanisms underlying gustatory perception in *Drosophila* have intensified (Freeman and Dahanukar, 2015; Shrestha and Lee, 2023; Chen and Dahanukar, 2020; Montell, 2009). However, the majority of these studies are focused on macronutrients, while limited exploration of taste perception of micronutrients, such as vitamins (Shrestha, Aryal, and Lee, 2023; Wu et al., 2021). Additionally, receptor expression exhibits spatial diversity, providing insights into specific feeding behaviors of fruit flies. Although some receptors are ubiquitously expressed, their functional deployment requires tissue-specific co-receptors, resulting in localized perception (McDowell, Stanley, and Gordon, 2022; Shrestha, Aryal, and Lee, 2023). This compartmentalization likely underlies observed feeding behaviors, with receptor localization patterns potentially reflecting specialized physiological roles (Kim H. et al., 2017).

#### 4.2.1 Ectopic taste receptors in gastrointestinal tract

Taste receptors have been identified in the GIT of insects (Toyam et al., 2023; Mang et al., 2023). Specifically, 12 *Grs* have been observed in the midgut endocrine cells (ECCs), organized into two distinct subpopulations with differential receptor profiles (Figure 6E). Notably, the sweet taste receptors Gr64a and Gr43a, along with several bitter taste receptors, were found in the ECCs, except for Gr66a and Gr32a, despite their wide expression in bitter GRNs. Furthermore, the co-localization of multiple taste receptors with regulatory peptides such as neuropeptide F (NPF), locustatachykinin (LTK), and diuretic hormone 31 (DH31) suggests that these receptors may play roles in sensing intestinal contents, regulating feeding behavior and gastrointestinal motility, and modulating intestinal osmotic pressure. Beyond ECCs, three taste receptors (Gr28b.b, Gr28b.c, and Gr28b.d) were expressed in non-ECCs of the midgut (Park and Kwon, 2011a). Gr28b.c is implicated in responding to various noxious stimuli in gustatory organs (Ahn and Amrein, 2023; Sang, Rimal, and Lee, 2019), while Gr28b.d is associated with heat sensing in the antennae (Capek et al., 2025), indicating potential non-nutritional sensory functions in the gut analogous to those in other organs.

#### 4.2.2 Ectopic taste receptors in nervous system

In *Drosophila*, several Gr28 genes are expressed in multidendritic neurons located in the abdomen, the putative hygroreceptor neurons of the arista, neurons linked with Johnston’s organ, peripheral proprioceptive neurons in the legs, and neurons within the brain of both larval and adult stages, as well as oenocytes. These GR genes are implicated in non-gustatory roles, including proprioception and hygroreception (Thorne and Amrein, 2008). Additionally, other studies further identified 18 GR genes and Ionotropic receptor gene Ir20a expressed in multidendritic neurons in the abdomen (Park and Kwon, 2011b; Koh et al., 2014).

Gr64a and Gr43a are expressed in the brain (Fujii et al., 2015), with Gr43a, as a fructose receptor, facilitating post-ingestion fructose perception in the brain (Miyamoto et al., 2012). Gr43a was also located in larval foregut neurons (Mishra et al., 2013), and its central activation may be mediated by the neuropeptide Corazonin (Miyamoto and Amrein, 2014). Importantly, synaptic connections also exist between enteric sNPF neurons and Gr43a neurons within the hypocerebral ganglion (HCG) in the foregut. Enteric sNPF neurons regulates the balance of sugar and yeast intake by modulating the neuronal activity of HCG Gr43a neurons (Yoshinari et al., 2024).

#### 4.2.3 Ectopic taste receptors in other organs and systems

Fourteen GRs exhibit olfactory system expression, including three sugar receptors (Gr5a, Gr64b, Gr64f), which potentially forming sugar receptors for gustatory functions or interacting with olfactory receptors to manifest novel functions (Fujii et al., 2015; Menuz et al., 2014). Most IRs also function as olfactory receptors within the fruit fly’s olfactory system, indicating the blurred boundaries of the insect chemosensation. Within the reproductive system, Gr43a expression has been observed in the uterus of *Drosophila* (Miyamoto and Amrein, 2014), while another study did not observe the expression of taste receptors in reproductive organs, but found the expression on the neurons innervating both male and female reproductive organs (Park and Kwon, 2011b).Additionally, flies with Gr28b mutations exhibit immunodeficiency, possibly due to altered feeding behavior impacting immune function *in vitro*, or a direct role *in vivo* immune responses (Ayres et al., 2008). In other Lepidopteran insects, taste receptors are expressed in the fat body of *Manduca sexta* (Koenig et al., 2015) and the Malpighian tubules of silkworm (Mang et al., 2016), though research on these expressions in *Drosophila* remains insufficient. Finally, it is crucial to note that current expression maps primarily derive from GAL4/UAS reporter systems, which may underestimate weakly expressing populations due to sensitivity thresholds and nonspecific background signals.

## 5 Taste and taste receptors in *C. elegans*

**FIGURE 7:**
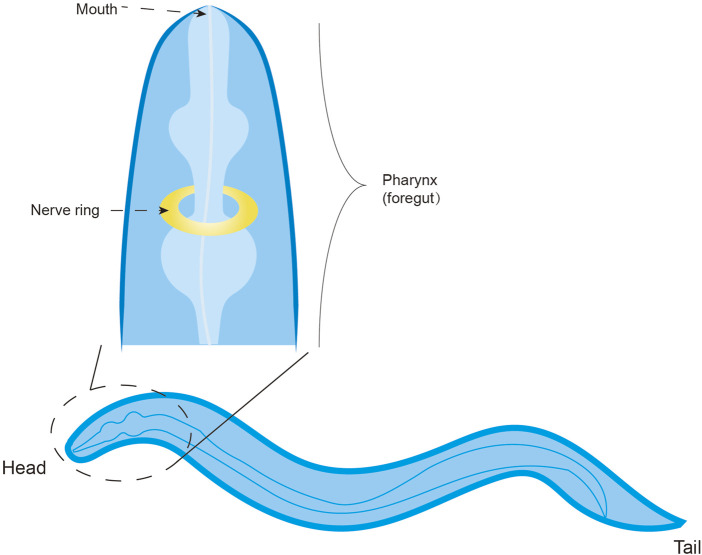
The taste system of *C. elegans*.

The nematode *Caenorhabditis elegans* possesses a fixed somatic cell count that differentiates into diverse tissues. The majority of its neurons are clustered near the pharynx, where their axonal projections coalesce into a circumpharyngeal nerve ring, functioning as the primary integrative neuropil (Hukema, 2006). Through laser ablation studies, five classes of gustatory neurons have been identified, each characterized by bilaterally symmetric pairs, with ASE neurons exhibiting the highest correlation (Etchberger et al., 2007) (Figure 7). Earlier studies have found that *C. elegans* can sense many water-soluble attractants, including salt, cAMP, amino acids and serotonin, mainly through ASE (Bargmann and Horvitz, 1991; Ward, 1973).

*C. elegans* expresses a repertoire of 1,341 genes encoding for chemosensory GPCRs, which display a partially overlapping and intersecting expression pattern within the sensory neurons responsible for gustatory perception (detection of water-soluble cues) and olfactory perception (detection of volatile cues) (Thomas and Robertson, 2008; Ferkey et al., 2021; Bargmann, 2006). Notably, these putative receptors are distributed not only within chemosensory organs but also across diverse interneurons, motor neurons, and non-neuronal cell types, suggesting their potential roles in detecting both environmental and internal cues (Vidal et al., 2018). However, only 244 of these genes have been functionally characterized. Given that some chemosensory receptors in *C. elegans* are not fully functionally differentiated between taste and smell, certain receptors may participate in the detection of both volatile odorants and soluble compounds. Moreover, some molecules, such as the receptor-type guanylyl cyclases, also act as taste receptors (Ortiz et al., 2009). Of particular interest is the plasticity of GPCR expression, especially during the dauer diapause stage induced by environmental cues, indicating that GPCR expression patterns may serve as a molecular signatures of the life history.

Additionally, *C. elegans* expresses the receptor LITE-1, a homolog of the insect seven-transmembrane receptor GR, distinguished by its inverted membrane topology with an intracellular N-terminus. Intriguingly, LITE-1 mediates phototransduction (Ghosh et al., 2021; Gong et al., 2016), expanding the sensory repertoire of this organism. Other chemosensory receptors include OTOP1, implicated in acid detection. *C. elegans* possesses eight OTOP genes, and single-gene mutants retain acid sensitivity, indicating functional redundancy (Li S, et al., 2023). While OTOP expression is primarily cephalic, transcripts of otpl-1, otpl-2, and otpl-5 have been detected in coelomocytes, warranting further investigation into their tissue-specific roles.

## 6 Conclusion and prospect

The emerging picture of ectopic taste receptors reveals an intricate sensory network extending far beyond canonical gustation. These molecular sentinels have evolved to serve important roles as systemic regulators of physiological homeostasis. Comparative analyses across species demonstrate both conserved functions (like nutrient sensing) and lineage-specific adaptations (such as osmoregulation in teleosts). The widespread distribution of taste receptors in non-gustatory tissues underscores their importance in fundamental biological processes including metabolism, immunity, and reproduction.

The functional roles of ectopic taste receptors, which deviate from canonical taste functions, in fact challenge the traditional notion of taste receptors as highly specialized, and instead reframe them within the broader context of chemosensation. Indeed, taste receptors in both gustatory and non-gustatory tissues, as well as other classes of chemoreceptors throughout the body, appear to operate via fundamentally similar mechanisms—all mediated physiological processes initiate from ligand-receptor interactions. Consequently, these studies provide strong experimental support for the recently proposed unified chemosensory theory, which elegantly integrates classical taste, olfaction, and chemical senses mediated by various receptors (including taste and olfactory receptors) under a single chemosensory framework without requiring further categorical distinctions (Mollo et al., 2022). This perspective may facilitate moving beyond the classical gustatory paradigm to develop research strategies for ectopic taste receptors that better reflect their fundamental physiological nature.

Beyond redefining the nature of taste receptors within the framework of a unified chemosensory theory, the key challenges in this field remain in elucidating the precise signaling mechanisms and functional consequences of extra-oral taste receptor activation. Future research should prioritize: (1) comprehensive receptor localization mapping using single-cell technologies, (2) structural characterization of receptor-ligand interactions, and (3) development of genetic tools for tissue-specific receptor manipulation. The therapeutic potential of targeting these receptors warrants exploration, particularly for metabolic disorders and immune modulation. Evolutionary studies comparing receptor repertoires across taxa could reveal adaptive patterns in sensory system diversification.

Technological advances in biosensors, organoids, and *in vivo* imaging will be crucial for probing receptor dynamics in physiological contexts. Integrating these approaches with systems biology may uncover novel receptor networks coordinating whole-body homeostasis. As the field moves beyond descriptive studies, mechanistic investigations should address how receptor signals are integrated with other sensory inputs and endocrine pathways. Ultimately, decoding the “taste receptome” may reshape our understanding of interoception and organism-environment interactions.
